# Extracorporeal Photopheresis Enhances the Frequency and Function of Highly Suppressive FoxP3^+^ Treg Subsets in Heart Transplanted Individuals

**DOI:** 10.1097/TP.0000000000005201

**Published:** 2025-03-19

**Authors:** Maria Mottola, Sara Bruzzaniti, Erica Piemonte, Maria Teresa Lepore, Andrea Petraio, Renata Romano, Antonella Castiglione, Lavinia Izzo, Francesco Perna, Chiara De Falco, Federico Brighel, Luigi Formisano, Maria Teresa Gravina, Marina Marino, Marisa De Feo, Giuseppe Matarese, Mario Galgani

**Affiliations:** 1 UOC di Medicina Trasfusionale, AORN dei Colli, Naples, Italy.; 2 Laboratorio di Immunologia, Istituto per l’Endocrinologia e l’Oncologia Sperimentale “G. Salvatore,” Consiglio Nazionale delle Ricerche, Naples, Italy.; 3 Unità di Neuroimmunologia, Fondazione Santa Lucia, Rome, Italy.; 4 Dipartimento di Medicina Molecolare e Biotecnologie Mediche, Università degli Studi di Napoli “Federico II,” Naples, Italy.; 5 UOSD Assistenza Meccanica al circolo e dei Trapianti, AORN dei Colli, Naples, Italy.; 6 Dipartimento di Medicina Clinica e Chirurgia, Università degli Studi di Napoli “Federico II,” Naples, Italy.; 7 UOC Biochimica Clinica, AORN dei Colli, Naples, Italy.; 8 Dipartimento di Neuroscienze e Scienze Riproduttive ed Odontostomatologiche, Università degli Studi di Napoli “Federico II,” Naples, Italy.; 9 Dipartimento di Cardiochirurgia e dei Trapianti, UOC Cardiochirurgia, AORN dei Colli, Naples, Italy.; 10 Dipartimento di Scienze Mediche Traslazionali, Università della Campania “L. Vanvitelli,” Naples, Italy.

## Abstract

**Background.:**

Extracorporeal photopheresis (ECP) has emerged as a prophylactic and therapeutic immunomodulatory option for managing acute rejection in heart transplants (HTx). The underlying mechanisms through which ECP exerts its immunomodulatory effects remain under investigation. Regulatory T cells (Treg) are a heterogeneous subset of immune lymphocytes that ensure the maintenance of tissue homeostasis, avoiding graft rejection. The transcription factor forkhead box protein 3 (FoxP3) is an essential molecular marker of Treg, acting as a “master regulator” of their genesis, stability, and functions. No study has investigated whether ECP impacts FoxP3 expression and its highly suppressive variants containing the exon 2 (FoxP3-E2), particularly in HTx.

**Methods.:**

In the current study, we recruited 14 HTx participants who had undergone ECP therapy. We explored the effect of in vivo ECP on CD4^+^FoxP3^+^ Treg frequency and in vitro suppressive function in 8 HTx participants before (T0) and after 3 (T1), 6 (T2), and 12 (T3) mo of treatment. As a control group, we included 4 HTx individuals who had not undergone ECP therapy.

**Results.:**

We found that ECP increases the frequency of CD4^+^FoxP3^+^ Treg subset with highly suppressive phenotype, including CD4^+^FoxP3-E2^+^ Treg. At functional levels, we observed that ECP treatment in HTx individuals effectively improves Treg suppressive ability in controlling the proliferation of autologous conventional CD4^+^ T lymphocytes.

**Conclusions.:**

Our findings collectively suggest that ECP exerts its immunomodulatory effects in HTx individuals by positively impacting the frequency and regulatory function of the FoxP3^+^ Treg compartment.

## INTRODUCTION

Heart failure continues to be a leading cause of morbidity and mortality worldwide, and cardiac transplantation is the gold standard for the treatment of heart failure refractory to standard medical therapy.^1^ However, allograft rejection episodes may occur because of the development of antidonor-specific cellular and antibody-mediated immune responses.^2^ In this context, clinical studies have demonstrated the beneficial effects of extracorporeal photopheresis (ECP) as a prophylactic and therapeutic immunomodulatory option to control acute rejection after solid organ transplantations, including heart transplantation (HTx).^3-6^ Experimental data have demonstrated the effectiveness of ECP, in combination with immunosuppressive drugs, in decreasing the risk of cardiac rejection.^3,7^ In ECP, mononuclear cells are exposed to ultraviolet-A (UVA) light after adding 8-methoxypsoralen (8-MOP), and treated cells are then reintroduced into the blood of the participants. It has been reported that 8-MOP molecules intercalate into the cellular DNA and, on subsequent exposure to UVA light, lead to the formation of DNA adducts, which in turn induce apoptosis in the treated cells.^8^ The beneficial effects of ECP are believed to result from multiple synergistic actions, and the overall mechanisms underlying its efficacy are currently being investigated. Compelling evidence reported that ECP exerts its immunomodulatory effects by promoting the differentiation of immature dendritic cells and the subsequent stimulation of tolerogenic T-cell lineages, particularly regulatory T cells (Treg).^9-11^ Treg constitute approximately 5%–10% of total CD4^+^ T cells and are characterized by the expression of the high-affinity interleukin-2 receptor alpha chain (or CD25) and the transcription factor forkhead box protein 3 (FoxP3).^12^ This immune cell population orchestrates the regulatory mechanisms aimed at suppressing effector T-cell responses, thus ensuring the maintenance of tissue immune homeostasis.^13^ Evidence supports the pivotal role of Treg in controlling solid organ transplant rejection and graft-versus-host disease; indeed, several therapeutic approaches are being used to enhance both their frequency and function.^14^ Many Treg subsets have been described nowadays with distinct phenotypes and suppressive capabilities. Based on the expression of CD45RA and FoxP3, Treg can be fractionated into 3 subgroups: the CD45RA^+^FoxP3^low^ resting Treg (fraction [Fr.] I) and the CD45RA^−^FoxP3^high^ activated Treg (Fr.II), which are both suppressive in vitro, and the CD45RA^−^FoxP3^low^ nonsuppressive Treg (Fr.III).^15^ In addition, different FoxP3 splicing isoforms have been described in human Treg^16^ to date; in particular, the FoxP3 splicing variants containing exon 2 (FoxP3-E2) are critical in conferring suppressive capability to Treg.^17-19^ Previous scientific evidence has shown the association between the frequency of CD4^+^CD25^+^ Treg and ECP treatment, including the study of several phenotypical markers associated with suppressive capacity, such as CD39.^20-22^ However, no studies have yet examined the frequency and suppressive function of CD4^+^FoxP3^+^ Treg in the context of ECP in HTx. Notably, the potential effects of ECP in the induction of highly suppressive Treg-containing Foxp3-E2 isoforms remain unexplored in this specific field.

Herein, we aim to investigate, at different time points, the potential immunomodulatory effects of ECP on FoxP3^+^ Treg subsets in individuals who have undergone HTx.

## MATERIALS AND METHODS

### Study Design and Patient Management

This is a prospective cohort study that recruited 14 HTx individuals who have undergone ECP treatment in addition to conventional immunosuppressive drugs, starting from 2020 to 2021 at the Azienda Ospedaliera Specialistica dei Colli (AORN) Monaldi – U.O.C. Medicina Trasfusionale (Naples, Italy). Heparinized whole blood samples were collected before (T0) and after 3 (T1), 6 (T2), and 12 (T3) mo of ECP treatment; blood withdrawal was collected before each ECP treatment session (**Figure S1, SDC,** http://links.lww.com/TP/D155). Among the 14 HTx individuals enrolled in the study, 2 participants died on T1 because of COVID-19, and 4 interrupted the ECP in our structure, for a total of 8 HTx-ECP individuals analyzed in the study. The decision to initiate ECP was made for prophylaxis in 3 HTx participants and as a therapeutic treatment in 5 HTx participants who had donor-specific anti-HLA antibodies and were at high risk for rejection (Table 1). As a control group, we included 4 HTx individuals who did not receive ECP therapy (HTx-CTR), recruited at about 36 mo from transplantation (T0), and then followed up for 6 mo (T2). All HTx patients were maintained on an immunosuppressive drug regimen during the studied period (see Table 1).

**TABLE 1.** Characteristics of HTx individuals treated with ECP (HTx-ECP) or not treated with ECP (HTx-CTR) analyzed in the study

Characteristics	HTx-ECP individuals	HTx-CTR individuals
No. of HTx analyzed	8	4
Age at study enrollment, y	28 ± 2.79	44.25 ± 4.17
Sex (male)	6	2
BMI	23 ± 1.11	25 ± 1.53
Therapeutic ECP	5	0
Prophylactic ECP	3	0
HTx and ECP interval, mo	20 ± 5.51	/
Immunosuppressive drugs		
Cyclosporine a and mycophenolic acid	2	1
Tacrolimus and mycophenolic acid	5	1
Everolimus and tacrolimus	1	1
Tacrolimus	0	1
Donor-specific antibodies		
Before ECP	5	/
After ECP T1	3	/
After ECP T2	2	/
After ECP T3	2	/
Cellular rejection		
After ECP T1	0	/
After ECP T2	0	/
After ECP T3	0	/

Data are reported using mean ± SEM in the case of numerical variables and absolute frequencies for categorical factors.

BMI, body mass index; CTR, control; ECP, extracorporeal photopheresis; HTx, heart transplant.

The demographic and clinical characteristics of all HTx individuals analyzed in the study are reported in Table 1. This study was approved by the Ethics Committee of the University of Naples “Federico II,” and all individuals gave their written informed consent.

### Extracorporeal Photopheresis

ECP was performed using the closed inline Therakos Cellex Photopheresis System (Therakos Inc) and the protocol was developed by the Pheresis Unit at the AORN Monaldi – U.O.C. Medicina Trasfusionale (Naples, Italy). The ECP protocol completed by each HTx patient was 43 ECP cycles during 12 mo; more in detail, the treatment included 2 ECP sessions on 2 consecutive days every week for the first month of therapy, then 2 ECP sessions every 2 wk for the second month of therapy and finally 2 ECP sessions every month. A complete blood count was performed before proceeding with the ECP procedure to assess leukocytosis or anemia and ensure that the patient is hemodynamically stable and has no signs of an acute infection. Vascular access was accomplished through a peripheral vein (%) and an indwelling tunneled central venous catheter (%). The ECP procedure is set to treat 1500–2000 mL of blood without interruption, with a flow rate of 30/35 mL/min, using a blood/anticoagulant sodium citrate (anticoagulant citrate dextrose solution-A) ratio between 10:1 and 16:1. At the end of the buffy coat collection, the operator adds 8-MOP; the software calculates the volume to be added. The machine completes the photoactivation process with UVA (1.5 J/cm^2^), and subsequently, the treated mononuclear cells are reinfused into the patient. Blood count analysis after reinfusion was performed.

### Blood Immunophenotype

Whole blood cells were analyzed with a clinical-grade hemocytometer to determine the absolute numbers of immune cells in each sample, and 100 μL of blood was used to evaluate, by flow cytometry, the percentage of immune cells, as previously described.^23^ Specifically, red blood cells were lysed using BD FACS lysing Solution 2 (BD Bioscience) and triple combinations of different human monoclonal antibodies were used to identify the different cell populations in the blood: FITC- and phycoerythrin (PE)-anti-CD3, PE- and PE-cyanine (PC) 5-anti-CD4, PC5-anti-CD8, PE-anti-CD16, PC5-anti-CD19, FITC-anti-CD45, PE-anti-CD56, PE-anti-CD45RA, and PE-anti-HLA-DR (all from Coulter Immunotech). Flow cytometry was performed on cells gated on CD45^+^—side scatter. Immunophenotypic analysis was performed with an EPICS XL flow cytometer (Beckman Coulter, Milan, Italy) using the Beckman Coulter XL System II software program.

### Peripheral Blood Mononuclear Cell Separation and Flow Cytometry Assay

Peripheral blood mononuclear cells (PBMCs) were isolated from the blood samples by centrifugation on a Ficoll-Histopaque density gradient, and plasma was stored at –80 °C until use. About 20 × 10^6^ PBMCs were frozen in fetal bovine serum plus 10% dimethyl sulfoxide to perform functional assays; the remaining aliquots of freshly isolated PBMCs were used to perform multiparametric cytofluorimetric analysis of Treg subsets. Specifically, PBMCs were stained with a mix of the following monoclonal antibodies: APC-H7 anti-human CD4 (BD Pharmingen, clone RPA-T4), FITC-anti-human CD45RA (Miltenyi Biotec, clone REA562), BB700 anti-human CCR7 (BD Horizon, clone 3D12), PECy7 anti-human CD25 (BD Pharmingen, clone M-A251), BV421 anti-human PD-1 (BD Horizon, clone EH12-1), antigen-presenting cell anti-human CD152/CTLA-4 (BD Pharmingen, clone BN13), PE anti-human FoxP3-All (BD Pharmingen, clone 259D/C7), or PE anti-human FoxP3-E2 (ThermoFisher Scientific, clone 150D/E4). Staining for intracellular factors was performed using the fixation and permeabilization FoxP3 buffer kit (BD Pharmingen), according to the manufacturer’s instructions. Samples were acquired using 2 lasers equipped with FACSCanto II (BD Bioscience) at least 3 × 10^4^ events in the lymphocyte gate. For the evaluation of positive events, fluorescence minus one control was used to set the gate; nonviable cells were detected by viability staining (BD Pharmingen). Cytofluorimetric analyses were performed using FlowJo Software (FlowJo, LLC). Gating strategies are reported in **Figure S2** (**SDC,** http://links.lww.com/TP/D155).

### Treg Expansion and Suppressive Assay

Tconv and Treg were isolated by positive magnetic separation (CD4^+^CD25^+^ Regulatory T Cell Isolation Kit, human, Miltenyi Biotech) by thawed PBMCs, according to the manufacturer’s instruction. Treg were expanded by 36-h stimulation with anti-CD3/CD28 microbeads (0.1 bead/cell) in Roswell Park Memorial Institute-1640 (Life Technologies) medium supplemented with 100 IU/mL penicillin, 100 μg/mL streptomycin (Life Technologies), heat-inactivated 5% autologous plasma and 100 IU/mL of human recombinant interleukin 2 (Roche) and 20 ng/mL of transforming growth factor β (Miltenyi Biotech). Tconv cells were cultured in media for 36 h and then stained with CellTraceViolet dye (ThermoFisher Scientific), according to the manufacturer’s instructions. Furthermore, CellTraceViolet^+^ Tconv cells were cultured for 96 h in round-bottomed 96-well plates (all from Becton Dickinson) alone or with different numbers of Treg (1:1–8:1 cell-to-cell ratios) with anti-CD3/CD28 microbeads (0.2 bead/cell), as previously described.^18^ Dilution of CellTraceViolet dye was used to measure the percentage of proliferating Tconv cells by flow cytometry. Results have been expressed as % of Treg suppression calculated as follows: 100*[(% of Tconv proliferation alone – % of Tconv-Treg ratio)/% of Tconv- proliferation alone].

### Statistical Analysis

Statistical analyses of data were performed using GraphPad Prism 9 software (GraphPad, CA). Demographic characteristics, clinical variables, and immune cells are reported in tables as mean ± SEM. Comparisons were performed using the paired nonparametric Friedman test or repeated measures-2-way ANOVA, as reported. A *P* value of <0.05 denotes statistical significance.

### Guidelines

This report complies with the Strengthening the Reporting of Observational Studies in Epidemiology Guideline for cohort studies.^24^

## RESULTS

### ECP Treatment Changes the Immune Profile in HTx Participants

First, we analyzed the effects of ECP on whole blood immune cell subsets from 8 HTx-ECP participants before and after 3 (T1), 6 (T2), and 12 (T3) mo of treatment. A progressive increase in the absolute number of leucocytes was found, with statistical significance reached after 12 mo of ECP treatment (Table 2). Regarding the lymphocyte population, we observed a decrease over time in the percentage of this cell subset on ECP treatment, although it did not reach statistical significance (Table 2); no differences were observed in the absolute number of lymphocytes (Table 2). A more specific analysis of the major lymphocyte subsets also revealed no significant differences in terms of both frequency and absolute number of CD3^+^, CD4^+^, CD8^+^, CD19^+^, and natural killer cells after ECP treatment (Table 2). Interestingly, we noticed a decrease in the memory compartments of T lymphocytes, including CD3^+^, CD4^+^, and CD8^+^ T cells, accompanied by an increase in the naive T-cell counterparts (Table 2). Furthermore, after ECP treatment, a significant decline was observed in the T-cell effector subsets, such as activating CD4^+^CD25^+^ T cells and cytotoxic CD8^+^HLA-DR^+^ T cells (Table 2).

**TABLE 2.** Absolute numbers and percentages of circulating immune cells in HTx participants before (T0) and after 3 (T1), 6 (T2), and 12 (T3) mo of ECP treatment

Immune populations	T0	T1	T2	T3	*P*
Leucocytes	3176 ± 335	3725 ± 456	3919 ± 200	5138 ± 1081	T0 vs T3*
Lymphocytes	1527 ± 114 50.31% ± 4.6%	1421 ± 154 39.78% ± 3.6%	1696 ± 324 42.58% ± 6.7%	1702 ± 323 37.61% ± 6.7%	NS NS
CD3^+^ cells	1170 ± 129 75.4% ± 4.2%	1099 ± 132 77.56% ± 3.6%	1252 ± 242 74.03% ± 4.6%	1283 ± 310 72.13% ± 4.6%	NS NS
CD4^+^ cells	394 ± 45 26.15% ± 2.6%	405.4 ± 45 29.93% ± 3.4%	401.9 ± 46 26.28% ± 2.4%	406.1 ± 68 25.58% ± 2.9%	NS NS
CD8^+^ cells	652 ± 98 41.55% ± 4.8%	611.8 ± 127 41.8% ± 4.5%	743 ± 196 41.4% ± 5.2%	768 ± 266 40.19% ± 5.6%	NS NS
CD19^+^ cells	140 ± 28 9.37% ± 2%	155 ± 35 10.6% ± 2.1%	190 ± 53 12.08% ± 3.1%	169 ± 31 10.93% ± 1.6%	NS NS
NK cells	197.4 ± 28 13.96% ± 2.6%	152 ± 31 10.91% ± 2.2%	223 ± 86 11.76% ± 3.5%	212 ± 48 14.75% ± 3.9%	NS NS
CD3^+^CD45RA^+^ cells	485 ± 81 30.43% ± 3.2%	622 ± 122 43.36% ± 6%	694 ± 129 42.96% ± 5.3%	831 ± 217 46.36% ± 5.5%	T0 vs T3* T0 vs T3*
CD3^+^CD45RO^+^ cells	684 ± 61 44.98% ± 3%	476 ± 51 34.2% ± 2.9%	610 ± 180 32.5% ± 4.1%	487 ± 104 28.54% ± 2.6%	T0 vs T3* T0 vs T3**
CD4^+^CD45RA^+^ cells	64 ± 14 4.06% ± 0.7%	121 ± 27 8.73% ± 2%	117 ± 20 8.77% ± 2%	149 ± 37 9.93% ± 1.9%	T0 vs T3* T0 vs T3**
CD4^+^CD45RO^+^ cells	329 ± 39 22.09% ± 2.7%	284 ± 29 21.19% ± 2.4%	285 ± 56 17.5% ± 1.5%	257 ± 49 16.35% ± 2.1%	NS T0 vs T3*
CD8^+^CD45RA^+^ cells	297 ± 58 18.66% ± 2.7%	419 ± 101 28.79 ± 4.7%	470 ± 119 27.84% ± 4.1%	561 ± 189 29.5% ± 5.1%	NS T0 vs T1, T2, T3*
CD8^+^CD45RO^+^ cells	# 355 ± 55 22.89% ± 3.2%	# 192 ± 50 13.01% ± 2.6%	# 273 ± 147 13.56% ± 5.2%	# 215 ± 86 10.11% ± 1.6%	T0 vs T1**; T0 vs T2, T3* T0 vs T1*; T0 vs T2, T3**
CD4^+^CD25^+^ cells	11 ± 3 0.7% ± 0.1%	9 ± 1 0.7% ± 0.1%	13 ± 5 0.7% ± 0.1%	7 ± 1 0.4% ± 0.1%	T0 vs T3* NS
CD4^+^HLA-DR^+^ cells	19 ± 6 1.3% ± 0.4%	23 ± 4 1.6% ± 0.2%	14 ± 4 % 0.7 ± 0.1	12 ± 4 0.7% ± 0.1%	NS NS
CD8^+^HLA-DR^+^ cells	33 ± 9 2.1% ± 0.5%	43 ± 20 2.6% ± 0.8%	24 ± 8 1.3% ± 0.3%	23 ± 9 1% ± 0.1%	T0 vs T2* T0 vs T3*

Data are expressed as mean ± SEM. The absolute number per mm^3^ of each cell population was calculated as follows: (% of a given cell population × absolute number of lymphocytes)/100.

* *P* < 0.05 by the paired Friedman test.

** *P* < 0.01 by the paired Friedman test.

ECP, extracorporeal photopheresis; HTx, heart transplant.

These findings suggest that ECP treatment modulates the blood immune cell profile, leading to a downmodulation of the effector memory responses in HTx-ECP participants.

### Increased Frequency of FoxP3^+^ Treg, Including the FoxP3-E2 Variants in HTx Participants on ECP Treatment

**FIGURE 1.**
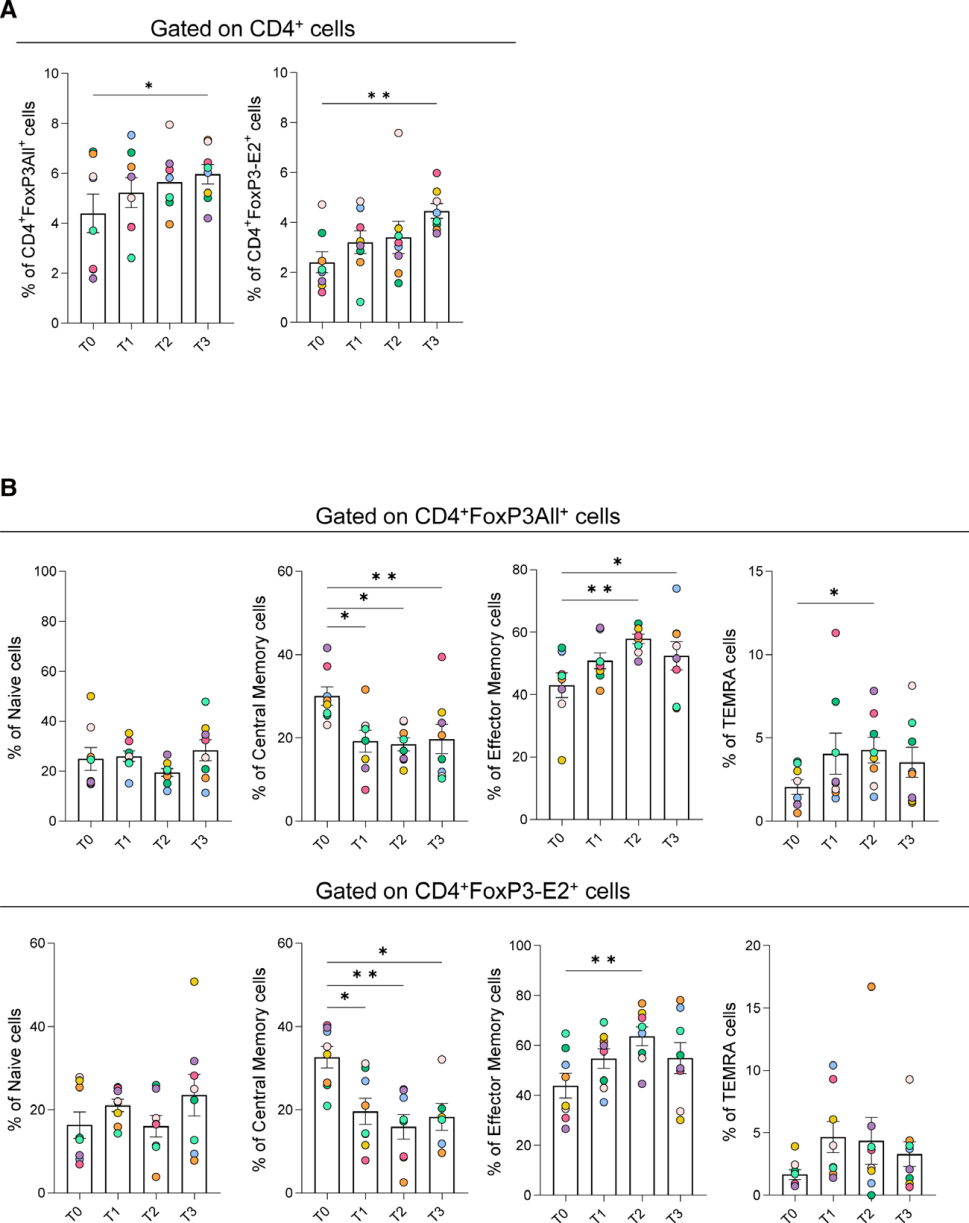
ECP treatment in HTx individuals increased the frequency of FoxP3^+^ Treg cell subsets, leading to an effector phenotype. A, Cumulative data showing the frequency of CD4^+^FoxP3-All^+^ Treg (left panel) and CD4^+^FoxP3-E2^+^ Treg (right panel) in HTx individuals before (T0) and after 3 (T1), 6 (T2), and 12 (T3) mo of ECP treatment. Data are expressed as mean ± SEM. Each symbol represents an individual. *P < 0.05, **P < 0.01 by the paired nonparametric Friedman test. B, Cumulative data showing the Treg differentiation subsets in CD4^+^FoxP3-All^+^ Treg (upper panels) and CD4^+^FoxP3-E2^+^ Treg (lower panels) before (T0) and after 3 (T1), 6 (T2), and 12 (T3) mo of ECP treatment in HTx individuals. Data are expressed as mean ± SEM. Each colored circle represents a different individual.*P < 0.05, **P < 0.01 by the paired nonparametric Friedman test. ECP, extracorporeal photopheresis; FoxP3, forkhead box protein 3; FoxP3-E2, FoxP3 exon 2; HTx, heart transplant; TEMRA, terminally differentiated effector memory; Treg, regulatory T cell.

Next, we assessed the impacts of ECP on Treg frequency measured by the expression of CD4^+^FoxP3^+^ cells, including those containing exon 2 variants (FoxP3-E2^+^ cells) in HTx participants. Cytofluorimetric analysis showed a progressive increase in the frequency of CD4^+^FoxP3All^+^ with statistical significance reached 12 mo from ECP treatment (Figure 1A, left panel). Concerning the expression of the specific higher suppressive isoforms FoxP3-E2, our data revealed that HTx participants increased the frequency of CD4^+^FoxP3-E2^+^ cells at all time points after ECP therapy (Figure 1A, right panel). No significant statistical differences were observed in the frequency of Treg subsets (both CD4^+^FoxP3All^+^ and CD4^+^FoxP3-E2^+^) on 6 mo of follow-up in HTx-CTR individuals (**Figure S3A, SDC,** http://links.lww.com/TP/D155).

Next, we evaluated the differentiation status in both CD4^+^FoxP3All^+^ and CD4^+^FoxP3-E2^+^ cells (naive and memory subsets). We found that ECP treatment in HTx participants decreased the frequency of central memory Treg and, on the other hand, increased the effector memory and the terminal effector memory (TEMRA) subsets (Figure 1B). Also, we analyzed the main molecules associated with regulatory function (ie, CTLA-4 and PD-1) in Treg subsets. No significant statistical differences were observed for the aforementioned markers on ECP therapy in HTx participants (data not shown).

These results suggest that ECP favors Treg increase, particularly those containing highly suppressive FoxP3 splicing variants.

### ECP Treatment Restored Treg Suppressive Function in HTx Individuals

**FIGURE 2.**
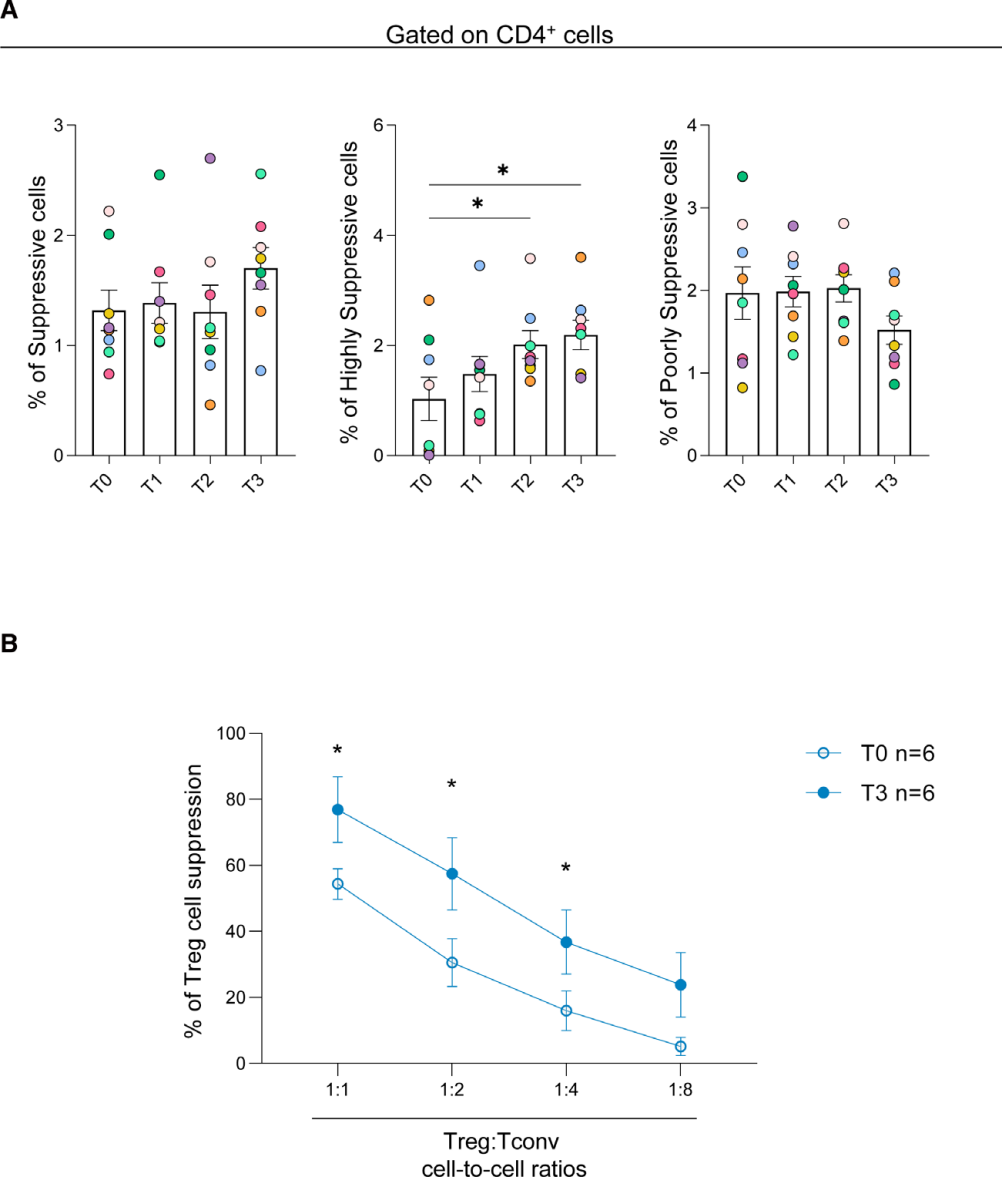
FoxP3^+^ Treg improved their suppressive phenotype and function on ECP treatment in HTx participants. A, Cumulative data showing the frequency of the 3 Fr. of CD4^+^FoxP3^+^ Treg in HTx individuals before (T0) and after 3 (T1), 6 (T2), and 12 (T3) mo of ECP treatment. Fr.I, suppressive; Fr.II, highly suppressive; Fr.II, nonsuppressive. Data are expressed as mean ± SEM. Each colored circle represents a different individual. *P < 0.05, **P < 0.01 by the paired nonparametric Friedman test. B, Cumulative data showing the suppressive function of freshly isolated Treg to control the proliferation of autologous Tconv cells (at different cell-to-cell ratios) before (T0) and after 12 (T3) mo of ECP treatment in HTx individuals. Data are expressed as mean ± SEM. *P < 0.05 by RM-2-way ANOVA. ECP, extracorporeal photopheresis; FoxP3, forkhead box protein 3; Fr., fraction; HTx, heart transplant; RM, repeated measures; Treg, regulatory T cell.

It has been reported that differential expression of FoxP3 and CD45RA molecules identify 3 Treg subsets with distinctive suppressive capabilities; phenotypical analysis, based on the expression levels of these markers, revealed that ECP increased the frequency of activated Treg with the high suppressive ability (Fr.II, CD45RA^–^FoxP3All^high^) compared with T0 (Figure 2A). Moreover, the percentage of suppressive Treg (Fr.I, CD45RA^+^FoxP3All^+^) and nonsuppressive Treg (Fr.III, CD45RA^–^FoxP3All^low^) were not significantly affected by the treatment over time (Figure 2A, middle and right). Notably, analysis of the Treg subsets in HTx-CTR individuals reported no significant statistical differences between T0 and T2 (**Figure S3B, SDC,** http://links.lww.com/TP/D155).

To confirm our results at functional levels, we isolated Treg from HTx participants before and after 12 mo of ECP treatment and cultured them with autologous CD4^+^ Tconv cells at different cell-to-cell ratios in the presence of T-cell receptor stimulation. Our data show that Treg from HTx participants are impaired in their suppressive capability before ECP treatment (Figure 2B); notably, Treg from ECP-treated participants recovered their functional ability to suppress Tconv cells in vitro (Figure 2B).

Collectively, these data indicate that ECP treatment in HTx increases the frequency of highly suppressive Treg subset while restoring their functional ability to regulate Tconv-cell proliferation in vitro.

## DISCUSSION

In this study, we report that ECP therapy changes the immune profile of T-cell subsets in HTx individuals and favors the increase of highly suppressive FoxP3^+^ Treg subsets, including those expressing the FoxP3-E2 variants. In parallel, we also found that in vivo ECP treatment enhances the suppressive capacity of Treg in vitro.

Over the last 30 y, ECP has been used as both prophylactic treatment and therapy of acute and chronic rejection after HTx.^25^ Despite its widespread clinical use, the precise mechanism of action of this treatment is still uncertain. Accumulating evidence suggests potential immunoregulatory properties of ECP therapy; for example, it has been observed that ECP induces apoptosis in circulating immune cells, thereby modulating antigen-presenting cells and favoring an increase in the frequency of Treg.^25^ Consistent with these observations, specific studies have reported increased percentages of Treg, identified as CD4^+^CD25^+^ T cells, in children with chronic heart and lung transplant rejection.^11,26^ In particular, Barten’s group analyzed several markers associated with functional subsets of Treg and found that ECP treatment increased the frequency of Treg expressing CD39, an ectoenzyme implicated in their suppressive functions.^20^ Although these studies have examined the Treg subsets on ECP therapy at phenotypical levels in transplant recipients, including HTx participants, none have specifically investigated the expression and regulation of the transcription factor FoxP3. Specifically, the modulation and expression of this crucial transcription factor on ECP treatment have been investigated exclusively within the framework of graft-versus-host disease.^27-29^ Thus, our findings extend previously published data, revealing that ECP therapy also improves the frequency of FoxP3^+^ Treg in HTx individuals. Notably, we found that ECP treatment increases the expression of FoxP3-E2 splicing forms in Treg over time, which are known to represent the variants that confer the highest suppressive capability to this immune cell subset.^17-19^ Through a comprehensive phenotypical analysis, we also observed that ECP therapy induces a change in the differentiation status of Treg, promoting their transition into effector memory counterparts, accompanied by an increase in the activated and more suppressive fraction of Treg (Fr.II Treg). These results suggest that in vivo ECP favors the increase of the Treg subset with suppressive phenotype, thus confirming regulatory populations as one of the main targets of the immunomodulatory role of ECP. Although previous research, including our own, has highlighted an increased circulating frequency of Treg with a suppressive phenotype in HTx individuals after ECP therapy, the impact of this treatment on the functional activity of Treg in vitro has remained unexplored. In this regard, we explored, for the first time, the functional capability of freshly isolated Treg before and after ECP. Our results revealed that Treg from HTx participants on ECP treatment displayed enhanced suppression of in vitro proliferation of autologous T-cell receptor–activated Tconv cells. Furthermore, our investigation also uncovered alterations in the immune profile of the main T-cell subsets, CD4^+^ and CD8^+^ T cells, among HTx individuals on ECP therapy, resulting in a shift in their differentiation status with a specific decline in the memory and effector counterparts. One possible explanation for the reduction of effector T-cell subsets could be attributed to the increased frequency and suppressive functions of FoxP3^+^ Treg, which in turn counteract the proliferation of activated T cells. This mechanism of action may elucidate the establishment of a tolerogenic environment that ultimately leads to improved clinical outcomes in HTx individuals, as evidenced by the stable clinical profiles, without graft rejection, of all participants in our study.

Although this is a single-center study, future research involving multicenter cohorts of ECP-transplanted individuals is necessary to gain a more comprehensive understanding of the potential association between elevated Treg and clinical responses to ECP. In conclusion, our research highlights the significance of analyzing FoxP3^+^ Treg, particularly FoxP3-E2 isoforms, as a specific immune biomarker indicative of ECP effectiveness and favorable transplantation outcomes.

## ACKNOWLEDGMENTS

The authors thank M. Montagna, S. De Simone, and all members of the IEOS-CNR for their technical support. **Figure S1** (**SDC,** http://links.lww.com/TP/D155) was developed using adapted images from Smart Servier Medical Art (https://smart.servier.com) and PowerPoint software (Microsoft Office 365).
